# 3D coronary dark-blood interleaved with gray-blood (cDIG) MRI

**DOI:** 10.1186/1532-429X-16-S1-P217

**Published:** 2014-01-16

**Authors:** Guoxi Xie, Bin Sun, Xiaoming Bi, Yutaka Natsuaki, Jing An, Qi Yang, Xin Liu, Hairong Zheng, Kuncheng Li, Debiao Li, Zhaoyang Fan

**Affiliations:** 1 Shenzhen Key Lab for MRI, Shenzhen Institutes of Advanced Technology, Shenzhen, Guangdong, China; 2 Biomedical Imaging Research Institute, Cedars Sinai Medical Center, Los Angeles, California, USA; 3 Union Hospital, Fujian Medical University, Fuzhou, Fujian, China; 4 Siemens Healthcare, Los Angeles, California, USA; 5 Xuanwu Hospital, Capital Medical University, Beijing, China

## Background

3D dark-blood MRI techniques have shown great potential in coronary plaque burden assessment [1]. However, a substantial variability in quantification could result from superficial calcification that often mimics part of lumen because of low signal. Recent work showed that gray-blood contrast can help to separate superficial calcification from lumen [2]. Thus, the purpose of this study was to develop a 3D coronary dark-blood interleaved with gray-blood (cDIG) MRI technique that potentially improve the visualization and quantification of coronary plaque.

## Methods sequence design

The cDIG method is developed based on a balanced SSFP sequence combined with a local reinversion (LocReInv) preparation as proposed by Botnar et al [3]. As with the LocReInv protocol, double inversion pulses are applied every two heartbeats and dark-blood images are collected in the first heartbeat. The novelty of cDIG is the acquisition of gray-blood images by utilizing the second heartbeat during which blood magnetizations have partially recovered. To improve gating efficiency, two independent respiratory navigators are used in two successive heartbeats, for dark-blood and grey-blood imaging, respectively.

## Imaging

The IRB approved study scanned 8 healthy volunteers (age 29 ± 9) on a 3T MR scanner (MAGNETOM Verio, Siemens, Germany). Imaging parameters included: TE/TR = 1.67/3.9 ms, Flip angle = 70, 0.81 × 0.81 mm 2 in-plane resolution (interpolated to 0.41 mm); 2.0 mm slice thickness for 3D cross-sectional imaging and it was interpolated to 1.0 mm for 3D in-plane imaging; 7/8 partial Fourier in phase direction; 822 Hz/pixel receiver bandwidth; 11~25 segments/heartbeat; SPAIR with a delay time of 180 ms for fat suppression. Cross-sectional imaging using LocReInv with the same scan parameters was performed for both image quality and signal intensity comparison. Wilcoxon signed rank test was conducted with p < 0.05 considered as significant.

## Results

**Figure 1:**
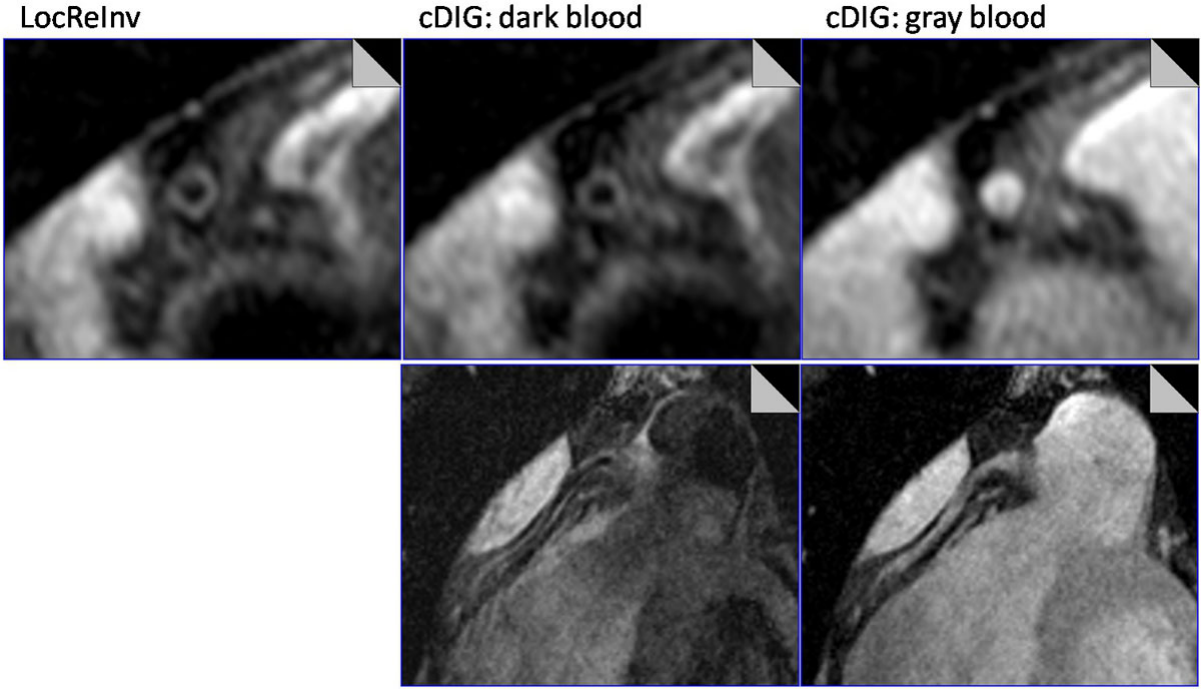
**Representative images of right coronary artery using the cDIG and LocReInv methods**.

All scans were successfully completed when using the cDIG and single-contrast LocReInv methods. Representative images are shown in Figure 1. Both vessel wall and lumen are clearly seen in dark-blood images. The values of SNR, CNR, wall thickness, lumen area as well as scan time are not statistically different between cDIG and LocReInv methods (Table 1). The cDIG method provides more information (gray-blood images), potentially facilitating the identification of calcified plaques and thus improving the accuracy of plaque burden assessment.

**Table 1:** Quantitative analysis results for image quality comparison between cDIG and LocReInv methods

Method	Vessel wall SNR	Lumen SNR	Epicardial fat SNR	Myocardium SNR	Wall/blood CNR	Wall/fat CNR	Wall thickness(mm)	Lumen area(mm2)	Acquisition Time (min)
cDIG (mean ± std)	19.4 ± 4.9	6.6 ± 3.0	9.6 ± 2.1	21.7 ± 6.6	12.8 ± 6.5	9.8 ± 3.8	1.4 ± 0.2	5.4 ± 1.6	8.1 ± 3.4

LocReInv (mean ± std)	20.2 ± 4.4	6.9 ± 2.8	10.8 ± 2.4	25.1 ± 8.2	13.3 ± 5.9	9.4 ± 2.9	1.4 ± 0.1	5.3 ± 1.9	7.0 ± 1.9

Wilcoxon test	ns	ns	ns	<0.05	ns	ns	ns	ns	ns

Note: ns - not significant

## Conclusions

A novel method for simultaneously obtaining coronary vessel wall and gray lumen images was proposed. In vivo experiments show dual contrasts were simultaneously acquired using the proposed method without compromising dark-blood contrast and scan time. This warrants further evaluation of cDIG on more volunteers and patients with coronary atherosclerosis.

## Funding

NHLBI HL38698, NIBIB EB002623, AHA-11POST7650043.
